# The usability and reliability of a smartphone application for monitoring future dementia risk in ageing UK adults

**DOI:** 10.1192/bjp.2024.18

**Published:** 2024-06

**Authors:** Graham Reid, Philip Vassilev, Jessica Irving, Triin Ojakäär, Liron Jacobson, Erin G. Lawrence, Jenny Barnett, Malika Tapparel, Ivan Koychev

**Affiliations:** Department of Psychiatry, University of Oxford, UK; Five Lives SAS, Tours, France; Five Lives SAS, Tours, France; and Nuffield Department of Clinical Neurosciences, University of Oxford, UK; Five Lives SAS, Tours, France; and Wolfson Institute of Population Health, Queen Mary University of London, UK; Five Lives SAS, Tours, France; and Department of Psychiatry, University of Cambridge, UK; Five Lives SAS, Tours, France; and Department of Medicine, University of Fribourg, Switzerland; Department of Psychiatry, University of Oxford, UK; and Five Lives SAS, Tours, France

**Keywords:** Clinical outcomes measures, dementias/neurodegenerative diseases, patients/service users, rating scales, clinical neurology

## Abstract

**Background:**

The rising number of dementia diagnoses and imminent adoption of disease-modifying treatments necessitate innovative approaches to identify individuals at risk, monitor disease course and intervene non-pharmacologically earlier in the disease course. Digital assessments of dementia risk and cognitive function have the potential to outperform traditional in-person assessments in terms of their affordability, accuracy and longitudinal tracking abilities. However, their accessibility and reliability in older adults is unclear.

**Aims:**

To evaluate the usability and reliability of a smartphone assessment of lifestyle and cognitive factors relevant to dementia risk in a group of UK-based older adults.

**Method:**

Cognitively healthy adults (*n* = 756) recruited through the Dementias Platform UK Great Minds volunteer register completed three assessments of cognitive function and dementia risk over a 3-month period and provided usability feedback on the Five Lives smartphone application (app). We evaluated cognitive test scores for age, gender and higher education effects, normality distributions, test–retest reliability and their relationship with participants’ lifestyle dementia risk factors.

**Results:**

Participants found the app ‘easy to use’, ‘quick to complete’ and ‘enjoyable’. The cognitive tests showed normal or near-to-normal distributions, variable test–retest reliabilities and age-related effects. Only tests of verbal ability showed gender and education effects. The cognitive tests did not correlate with lifestyle dementia risk scores.

**Conclusions:**

The Five Lives assessment demonstrates high usability and reliability among older adults. These findings highlight the potential of digital assessments in dementia research and clinical practice, enabling improved accessibility and better monitoring of cognitive health on a larger scale than traditional in-person assessments.

Dementia represents a significant global health challenge, with implications for individuals, families and healthcare systems. The rising prevalence of the condition, however, has recently been countered by the imminent prospect of disease-modifying therapies entering clinical practice (i.e. amyloid clearance immunotherapies).^1^ That said, these therapies carry a significant risk of side-effects and can be prohibitively expensive. For instance, 1 in 10 clinical trial participants develop cerebral oedema or brain haemorrhage while taking lecanemab,^2^ which has now been approved by the US Food and Drug Administration. Health economic analyses conducted in the European Union indicate that if lecanemab were given to all eligible individuals, its cost alone would account for half of the Union's current medication budget.^3^ The high costs of both dementia care and its therapies highlight the need to emphasise both early detection and non-pharmacological approaches to reducing disease burden in its preclinical and prodromal stages. This shift in focus requires sensitive and scalable means of identifying dementia risk.

## The preclinical window of opportunity

The nature of dementia pathogenesis makes the condition a suitable target for interventions geared at primary and secondary prevention. Risk factors for dementia accumulate across the lifespan, and include genetic predispositions, medical conditions and environmental exposures. Twelve modifiable factors have been found to contribute to up to 40% of worldwide dementia cases.^4^ For example, early-life factors such as low educational level may set the stage for increased susceptibility to dementia later in life. As individuals age, additional factors, such as cardiovascular disease, diabetes and sedentary lifestyle, can further compound risk.^5^ Furthermore, dementia pathology tends to emerge decades before a formal clinical diagnosis.^6^ This extended pre-symptomatic period provides a crucial window of opportunity for secondary dementia prevention, enabling the identification of high-risk individuals for early intervention before disease onset. However, the current healthcare system is under-resourced and ill-equipped to perform large-scale dementia risk screening or intervention, given the increasing number of cases and the difficulty in identifying individuals in early stages of the disease. Dementia risk factors are also often complex and interrelated, necessitating a comprehensive assessment to accurately evaluate an individual's risk profile, which is resource-intensive and challenging to implement without technology that can track multiple and interacting risk factors over time.^7^

Digital healthcare technologies, particularly smartphone applications (apps), may overcome some of the barriers associated with traditional in-person dementia assessments.^8,9^ These digital technologies offer some advantages over traditional methods. In the preclinical stages of dementia, cognitive decline often occurs gradually, and early symptoms may be subtle.^10^ Regular monitoring, facilitated by longitudinal digital healthcare technologies, may therefore become essential for detecting subtle cognitive changes indicating early signs of dementia. By leveraging advanced technologies, these digital assessments could effectively track and identify preclinical individuals in ways that surpass conventional methods employed by clinicians.^11^ This capability has the potential to enhance earlier detection and intervention, which are crucial for effective management of the global burden of the disease.

## Validated assessment tools

To maximise the effectiveness of remote digital assessments for dementia risk prevention, it is crucial that they are feasible and use validated and reliable tools that measure constructs with demonstrable relevance to dementia risk.^12^ Promising cognitive markers of early dementia risk include assessments of verbal fluency, verbal and visual episodic memory, executive function and processing speed. For instance, studies have shown that individuals with very mild dementia due to Alzheimer's disease tend to generate fewer words and word clusters compared with healthy older adults.^13^ Poorer performance on both verbal and visual memory tests has also been found to predict future dementia up to 10 years before a formal diagnosis.^14,15^ Additionally, executive functioning seems to strongly discriminate healthy individuals from those in early stages of Alzheimer's disease in which deficits in inhibition tend to outperform other cognitive domains in discriminability metrics.^16,17^ And last, although domain-specific testing is important, evidence also suggests that a general measure of processing speed is a useful indicator of possible neurodegeneration, as people with suspected dementia show longer response times in tests.^18^ Together, it should be noted that such composite testing that includes measures of executive function, episodic recall, verbal fluency and processing speed have been noted for their sensitivity in assessing deviations from normal cognition in asymptomatic amyloid-positive individuals, further highlighting their utility in tracking cognitive changes in preclinical individuals.^19,20^

## Enhancing long-term engagement

Beyond the validity of cognitive assessments, the ultimate success of digital dementia risk assessments will rely on creating user-friendly platforms that cater to older adults with varying levels of technological proficiency. The ideal assessment requires minimal supervision, can be completed remotely and provides clear instructions and intuitive interfaces, which maximise user engagement for repeat assessments and continuous monitoring. In doing so, remote dementia assessments may become mainstream tools not only for tracking cognitive changes and identifying individuals at risk for dementia, but potentially even for evaluating the effectiveness of emerging dementia treatments. In the present study, we aimed to assess the usability and reliability of a smartphone app in older adults developed by Five Lives in collaboration with Dementias Platform UK (DPUK) at the University of Oxford.

## Method

### Participants

The study recruited 756 participants aged between 50 and 79, through the DPUK Great Minds volunteers register.^21^ Participants had to be fluent English speakers and have no reported diagnosis of dementia or mild cognitive impairment. The only exclusion criterion was the lack of access to a suitable mobile device. Participants were approached based on their performance on the Cambridge Neuropsychological Test Automated Battery (CANTAB) Paired Associates Learning (PAL) task (a part of the Great Minds test battery; cambridgecognition.com/) being within 1.5 s.d. of the age-, gender- and education-adjusted mean. The rationale for this stratification was to ensure that this pilot study would gather data from participants with cognitive abilities representative of their age group.

### Ethics

The authors assert that all procedures contributing to this work comply with the ethical standards of the relevant national and institutional committees on human experimentation and with the Helsinki Declaration of 1975, as revised in 2008. All procedures involving human subjects were approved by North West – Haydock Research Ethics Committee (21/NW/0053). Participants provided written informed consent before any study procedures were carried out.

### Materials

The Five Lives risk assessment was delivered through the Five Lives smartphone app (https://www.fivelives.health/app?). The assessment covers four main areas: basic demographic information, self-reported health and lifestyle risk factors for cognitive decline, self-reported memory concerns and five cognitive tests measuring distinct aspects of cognition that are known to measure dementia-related cognitive impairment.

#### Self-reported health and lifestyle risk factors

A transcript of the full Five Lives questionnaire is available in Supplementary material 1, available at https://dx.doi.org/10.1192/bjp.2024.18. The questionnaire has three modules. ‘About You’ gathers demographic information, including the participant's date of birth, highest level of education, occupational status, height, weight, English proficiency, first-degree relatives with dementia, living area and asks two questions regarding subjective memory concerns. ‘Your Health’ asks questions about the participant's medical conditions known to be relevant to cognitive decline and any medication that they are taking. ‘Your Lifestyle’ asks questions about the participant's diet, exercise, mental activity, sleep, mood, smoking, alcohol consumption and working with toxic materials.^22^

#### Cognitive tests

The Five Lives assessment included five tests that measured distinct cognitive domains: semantic and phonemic fluency (‘Snap’), verbal memory (‘Breeze’), visuospatial memory (‘Cast’), executive function (‘Swift’) and speed of processing and cognitive control (‘Twist’) (Table 1). A detailed description of the cognitive tests, as well as representative images of the user interface, can be found in Supplementary material 2.

**Table 1:** A description of the Five Lives cognitive tests

Test name	Instruction	Analogous task	Cognitive function	Scoring	Outlier handling
Snap	List as many words as possible from a given category (animals, fruits, vegetables) or beginning with a given letter (f, c)	Verbal fluency^19^	Semantic fluency, phonemic fluency	Number of words produced	Not applicable
Breeze	Recall 16 different words from unique categories, with and without memory cues	Free and cued recall^20^	Verbal memory	Number of words freely recalled. Proportion of words recalled that were cued	Exclude responses where reaction times were more than 3 s.d. from the mean for all participants
Cast	Recall the location of different shapes on a 4 × 4 or 5 × 5 grid of white squares	Paired associates learning^21^	Visuospatial memory	Number of correctly recalled shape locations	Exclude responses with reaction times 3 s.d. from the mean for all participants
Swift	Say the actual colour of a presented word, and not the colour the word refers to or *vice versa*	Stroop^22^	Executive function	Median response time for correct answers where the colour and meaning of the word do not match	Exclude responses where reaction times were more than 3 s.d. from the mean for that participant
Twist	Tap on 20 consecutive numbers and/or letters arranged in a random visual array as quickly as possible	Trail Making A and B^23^	Processing speed	Total time until correct across three trails	Exclude trails with response times 3 s.d. from the mean for all participants

### Procedure

Participants were instructed to download the app on their preferred mobile device (smartphone or tablet) and complete the Five Lives assessment three times: at baseline (assessment 1), 2 weeks later (assessment 2) and 3 months from baseline (assessment 3). Each assessment session required completion of the Five Lives questionnaire and five cognitive tests. The response windows for each assessment lasted for 2 weeks. Reminders to complete the assessment were sent via email on the initial release date and 1 week later. Following assessment 1, participants were asked to complete a short survey online about their experience with the app. Participants were not given a dementia risk result from their assessment answers.

### Statistical analyses

#### Assessment acceptability

Acceptability of the app was quantified as assessment completion rates and time taken to complete each assessment. We also asked participants to complete 10-point Likert scale questionnaires on the app's ease of use, asking ‘How would you rate the app in terms of its ease of use? (1 = very difficult; 10 = very easy)’, the length of the assessment, asking ‘How did you find the length of the assessment? (1 = very long; 10 = very short)’ and the difficulty of the assessment, asking ‘How did you find the difficulty of the assessment? (1 = very difficult; 10 = very easy)’. Open-ended qualitative questions were also included to assess which aspects participants enjoyed and which features they think could be improved.

#### Test–retest reliability

We calculated the intraclass correlation coefficient (ICC)^23^ to assess the test–retest reliability of the composite ‘pillar of health’ scores of sleep, physical activity, stress, mental stimulation and diet across the three assessments. Each pillar score was calculated from multiple questions, with a total possible score of 1000. For a full description of the calculations for each pillar, see Supplementary material 3.

#### Cognitive test performance

Table 1 presents the scoring methodology and outlier handling for each test. Linear regressions were performed to examine the effects of age, gender and having attended college or university on the performance on each cognitive test. Repeated-measures analyses of variance (ANOVAs) were conducted to identify practice effects due to multiple assessments. To avoid false-negative results, thereby overlooking practice effects, we did not apply a family-wise error rate correction. Test–retest reliability between assessments was assessed using ICCs. As participants were drawn from the Great Minds register, they had all undergone previous external testing as part of the CANTAB. Therefore we also correlated participants’ scores on the ‘Cast’ test with their scores on the CANTAB's PAL task, which is considered a gold standard measure of visuospatial memory.^21,24^

#### Clustering analysis based on lifestyle scores

To classify participants into different lifestyle groups based on their self-reported lifestyle scores, we calculated a composite lifestyle score by averaging the five ‘pillars of health’ scores: diet, physical activity, sleep, mental stimulation and mood. We hypothesised the existence of three clusters, with low, medium and high lifestyle scores. We used hierarchical clustering to analyse lifestyle scores from the three assessments (without averaging across time points) using squared Euclidean distance as the dissimilarity measure. This was done to enhance the interpretability of the results and to avoid a large number of comparisons that would inflate type I error rates. Visual inspection of the dendrogram informed the number of clusters extracted. Missing values were removed. To characterise each of the extracted clusters in terms of lifestyle score, we employed a mixed-effects linear model, with lifestyle score as the dependent variable and assessment time point and lifestyle cluster as the independent variables. We accounted for the repeated measures within each participant. Repeated-measures ANOVAs were used to assess whether participants from different lifestyle clusters performed differently on the five cognitive tests.

## Results

### Sample demographics

The majority of the study sample were female (67.46%) and the mean age was 65 years (s.d. = 7.9). The mean weight was 75.51 kg (s.d. = 16.27) and the mean height was 167.78 cm (s.d. = 9.11). The largest proportion of participants did not smoke (63.1%), were educated to undergraduate university degree level (36.51%), were retired (66.67%) and lived in rural areas (44.44%). Most participants perceived their memory to be similar to others in their age group (82.67%), but also as being worse than a few years ago (51.72%). For a full description of the participants’ demographics, see Supplementary material 4.

### Self-reported risk factors

Participants reported several risk factors for dementia (see Supplementary material 5 for the full results). Among the respondents, 34.43% reported having at least one first-degree relative with dementia, 15.64% reported high blood pressure, 14.84% reported high cholesterol levels, 5.57% reported being diagnosed with diabetes, 1.44% reported having had a stroke, 28.66% reported hearing problems and 43.92% reported visual problems.

### Assessment acceptability

Most participants completed the assessments within the required window period of 2 weeks. Assessments 1, 2 and 3 had 95, 92 and 88% completion rates respectively. Overall, 87, 79 and 81% completed assessments 1, 2 and 3 respectively within 24 h or less. The median completion times were 31, 35 and 39 min for assessments 1, 2 and 3 respectively. On a scale of 1 to 10, the Five Lives app was rated as easy to use (mean 8.7, s.d. = 1.6) and participants rated the assessments as short (mean 6.7, s.d. = 1.9) and easy (mean 6.9, s.d. = 1.8) to complete. Qualitative analyses revealed that participants found ‘remembering’ and ‘shapes’ most difficult in the assessment, indicated by a word frequency of 4 and 3% respectively. Participants enjoyed the ‘cognitive’ (2% word frequency) parts of the ‘tests’ (7% word frequency) but indicated that future versions of the app could have better ‘instructions’ (2% word frequency). A fuller summary of responses to the eight Likert-style questions can be found in Supplementary material 6.

### Test–retest reliability

#### Lifestyle questionnaire

Each pillar of health demonstrated substantial to strong reliability, with ICCs of 0.77, 0.79, 0.89, 0.84 and 0.81 for sleep, physical activity, stress, mental stimulation and diet respectively (Supplementary material 7).

#### Cognitive tests

Good-to-substantial reliability was demonstrated for the semantic and phonemic fluency tests (Snap: 0.75 and 0.71), the executive function test (Swift: 0.78) and the speed of processing and cognitive control test (Twist: 0.60). Moderate reliability was demonstrated for the verbal memory test (Breeze: 0.45 for freely recalled words and 0.40 for the proportion of recalled cued words) and for the visuospatial memory test (Cast: 0.56). Visualisations of the distributions for each test and inter-assessment correlations can be found in Supplementary material 8.

### Effect of demographics on cognition

Linear regression analyses found effects of age, gender and education on cognitive testing (Table 2). Although a negative relationship between age and performance was found on all cognitive tests, gender differences were only found for tests measuring verbal memory and verbal fluency. Participants who had completed college or university also performed better on verbal tests. There were no significant effects of gender or higher education for the Cast, Swift or Twist tests.

**Table 2:** Linear regressions of performance on the Five Lives cognitive tests as a function of age, gender and educational level

Test	*n*	Metric	Adjusted *R*2	Age, β	Male, β	Education, β
Breeze	458	Freely recalled words	0.08	−0.05***	−0.73***	0.85**
		Proportion of cued words recalled	0.09	0.01***	0.05***	−0.05**
Cast	464	Correct answers	0.08	−0.25***	−0.44	−0.163
Snap	767	Words generated (initial letter)	0.13	−0.12***	−0.80**	1.67***
		Words generated (semantic category)	0.18	−0.10***	−1.25***	0.98***
Swift	767	Congruent matches (ms)	0.10	22.00***	81.55	−11.86
		Incongruent matches (ms)	0.11	22.02***	58.33	−69.59
Twist	767	Completion time (ms)	0.09	1580.1***	2293.8	−7371.9

**P < 0.01, ***P < 0.001.

### Practice effects

ANOVAs revealed a pattern of improved performance after completing each test once (Table 3). Improvements on the Breeze and Cast tests were sustained at the 3-month assessment (*P* < 0.001). Furthermore, there were statistically significant increases in performance from the 2-week to 3-month assessments for measures of semantic fluency in the Snap test and executive function in the Swift test, and a statistically significant decrease in performance for phonemic fluency in the Snap test (*P* < 0.001). Additionally, there were improvements in performance from 2 weeks to 3 months on the executive function test (Swift; *P* < 0.05) and the speed of processing test (Twist; *P* < 0.005).

**Table 3:** ANOVA results for practice effects on Five Lives cognitive test performance

Test	Metric	*F*	d.f.	Mean change between assessments, s.e.		
				2–1	3–1	3–2
Breeze	Freely recalled words	72.01	2, 294	1.67 (0.15)***	1.35 (0.15)***	−0.32 (0.15)
	Proportion of cued words recalled	62.07	2, 294	−0.10 (0.01)***	−0.09 (0.01)***	0.01 (0.01)
Cast	Correct answers	10.56	2, 199	1.25 (0.42)***	1.90 (0.42)***	0.65 (0.42)
Snap	Words generated (initial letter)	30.68	2, 463	0.59 (0.13)***	−0.40 (0.13)	−0.99 (0.13)***
	Words generated (semantic category)	83.97	2, 476	1.54 (0.19)***	2.45 (0.19)***	0.91 (0.19)***
Swift	Congruent matches (ms)	16.71	2, 483	−57.8 (17.9)**	−103.3 (17.9)***	−45.4 (17.9)*
	Incongruent matches (ms)	21.18	2, 483	−77.7 (19.8)***	−128.2 (19.8)***	−50.5 (19.8)*
Twist	Completion time (ms)	88.21	2, 461	−17 957 (1884)***	−24 079 (1884)***	−6123 (1884)**

*P < 0.05, **P < 0.01, ***P < 0.001.

### Validity of visuospatial memory assessments

The number of errors/total rounds completed on the Cast test correlated positively with the adjusted total number of errors on the CANTAB-PAL test (ρ = 0.36, *P* < 0.001; Supplementary material 9).

### Relationship between lifestyle and cognition

#### Hierarchical clustering analysis

A hierarchical clustering analysis produced a dendrogram with three distinguishable clusters (Supplementary material 10). Across the three assessments, cluster 1 had the highest average lifestyle score, followed by cluster 3 and then cluster 2 (Supplementary material 11). There was a significant main effect of lifestyle cluster, with cluster 2 (β = −314.268, *P* < 0.001) and cluster 3 (β = −131.643, *P* < 0.001) showing lower lifestyle scores compared with cluster 1. There was also a significant interaction between the second assessment and cluster 2 (β = −22.777, *P* = 0.015), and the third assessment and cluster 3 (β = 12.767, *P* = 0.029), indicating that lifestyle scores in the ‘high’ cluster decreased slightly from the first assessment to the last, whereas lifestyle scores in the ‘medium’ cluster increased in the last assessment relative to the first (Supplementary material 11).

#### Cluster differences in cognitive performance

ANOVAs showed no significant differences in cognitive performance between lifestyle clusters at the initial and final assessments, except for Breeze scores at the final assessment (Table 4). Nevertheless, subsequent *post hoc* analyses using the Tukey honestly significant difference (HSD) test on the Breeze scores did not identify any significant differences among the lifestyle clusters. This was likely due to the smaller sample size in the ‘low’ lifestyle score cluster, where only 31 participants completed the Breeze test (compared with *n* = 121 and *n* = 179 in the ‘high’ and ‘medium’ clusters respectively).

**Table 4:** ANOVAs testing the effect of lifestyle cluster on Five Lives cognitive test performance

Test	Metric	d.f.	ANOVA *F*-value		
			Score at initial assessment	Score at final assessment	Percentage increase from assessment 1 to assessment 3
Breeze	Freely recalled words	2	2.483	3.530*	1.530
	Proportion of cued words recalled	2	3.722	2.847	0.462
Cast	Correct answers	2	0.218	0.416	1.248
Snap	Words generated (initial letter)	2	0.247	0.125	1.041
	Words generated (semantic category)	2	0.358	0.532	0.968
Swift	Incongruent matches	2	0.606	0.032	0.614
Twist	Completion time	2	0.459	0.083	1.743

* *P* < 0.05.

## Discussion

The aim of this study was to assess the usability and reliability of a digital assessment of dementia risk and cognitive function in healthy older adults. The findings revealed that older adults from a research-interested register found the cognitive assessments quick and easy to complete. Cognitive scores showed normal-to-near-normal distributions, with variable reliabilities across different tests. Although cognitive scores did not correlate with participants’ self-reported lifestyle, we found that age had a significant impact on all cognitive tests, but gender and education only influenced tests concerning verbal ability.

This study adds to the existing literature by demonstrating that older adults in our study have positive attitudes towards remote dementia assessments, as evidenced by their favourable ratings of the user experience.^25^ Furthermore, we expand on previous research that has solely focused on cognitive assessments, by additionally assessing both medical and lifestyle risk factors simultaneously.^26^ By combining risk factors and cognitive outcomes, our approach surpasses the limitations of isolated assessments and holds the potential for more enhanced risk identification.

### Protective and risk factors

Regarding the cognitive tests we used, we found a female and education-related advantage in verbal fluency and verbal episodic memory. These findings align with existing literature showing that females tend to have stable verbal fluency scores between the ages of 40 and 80, whereas males tend to show steeper decline.^27,28^ As with verbal fluency, our results also support the existing evidence that females have a slight advantage in verbal recall, as shown in a recent meta-analysis.^29^ As for education, it has been shown in other studies to predict verbal fluency^30^ and verbal memory,^31^ but not visuospatial skills,^32^ with limited research on executive function and processing speed. It is possible that the education system's emphasis on language-based instruction and assessment contributes to enhanced verbal abilities, perhaps leading to domain-specific effects of education on older adults’ cognitive abilities.

In contrast to education, we found that age was associated with decreased cognitive performance across all tests, which is consistent with literature suggesting that age is the strongest risk factor for cognitive impairment and dementia. Throughout the lifespan, the accumulation of neurodegenerative risk factors, including brain pathology and cerebrovascular risk factors, affects cognitive abilities in a progressive manner.^33^ Our study found no correlation between participants’ self-reported lifestyle clusters and cognitive abilities, which is to be expected as dementia risk accumulation in the earlier lifespan is likely to affect future cognitive abilities. Although our study collected data on lifestyle and cognition over a 3-month period, tracking lifestyle risk and future cognitive abilities over longer time frames would allow more realistic modelling of the relationship between lifestyle and cognition throughout the lifespan. There are already apps, such as the Cardiovascular Risk Factors, Aging and Incidence of Dementia (CAIDE) Risk Score app, available to model 20-year dementia risk from midlife vascular factors, which form a useful template for future research.^34^ However, they lack the capability to directly measure cognitive changes, a key feature of our assessment.

### Practice effects

As would be expected from existing research, our study showed that multiple testing sessions result in enhanced performance in subsequent cognitive assessments.^35^ Practice effects occur due to increasing familiarity with cognitive tasks and improved strategies developed through repeated testing over time. Initial assessments can be challenging or anxiety-provoking, leading to below-expected performance that regresses to the mean in subsequent tests. Research has shown that modelling practice effects, or the lack thereof, can serve as a useful marker of early cognitive decline since attenuated practice effects have been shown to predict older adults’ progression to Alzheimer's disease, as well as to differentiate those with dementia from healthy age-matched controls.^36^

### Distribution of test scores

Just like an individual's ability to learn with practice, the distribution of test scores can also be a useful indicator of cognitive impairment.^37^ Importantly, in our study, cognitive test scores followed normal or near-normal distributions. This suggests that the tests effectively capture the range of cognitive abilities in the population under study and that they have the potential to accurately identify individuals with cognitive impairment. This highlights the utility of digital assessments for tracking dementia risk, as individual scores can be compared with normal distributions to gain relative insight into a participant's cognitive abilities. For instance, standardised scores derived from the normative distributions can identify significant deviations that might indicate cognitive impairment. With a sample of over 700 participants, we have improved on the ability to capture normative scores since previous research has often used much smaller samples.^26^ By including older adults with diverse educational backgrounds, current health conditions and sociodemographic characteristics, we aimed to avoid skewed or incomplete representations of the population's cognitive abilities, thereby supporting the development of more equitable and inclusive assessment practices.

### Limitations

This study has four main limitations to consider. First, our participants were a self-selected group of older adults who were signed up to a research-interested register. These older adults will likely have been more technologically literate than average as they were able to use information technology at the start of the study (e.g. downloading and interacting with a smartphone app). This factor will limit the broader representativeness of the study sample. Additionally, most participants were female, and we did not gather data specifically on participants’ ethnic or socioeconomic backgrounds, aside from educational level. It is well-known that populations engaged in research lack diversity in respect to these characteristics. Considering that factors such as ethnicity, socioeconomic status and gender can influence both dementia risk and ease of using smartphone technology, future studies exploring digital apps in dementia should aim to include a more diverse sample, representing varied genders, ethnicities and socioeconomic backgrounds. Second, standardised testing conditions between participants, and between assessment time points, can be challenging when using remote assessments. Factors such as screen size, device type and environmental distractions may have influenced participants’ responses and performance on cognitive assessments and risk questionnaires. Third, cognitive function and human behaviour are often influenced by the social environment, including social engagement, non-verbal cues and emotions derived from the surroundings. Assessing cognitive abilities in isolation through smartphones may not fully reflect older adults’ true cognitive abilities. This lack of personal context may have influenced the reporting of lifestyle factors, which are typically influenced by social desirability, response bias and expectancy effects when in-person assessments are conducted. Finally, we note that the task completion rates decreased as the study progressed. This highlights the general challenge of user engagement across digital platforms and services. Our participant and public involvement work that led to the study showed that ageing adults say they are more likely to engage with dementia risk assessment if it is linked to treatments. We therefore hypothesise that engagement can be maintained through incorporating such digital risk assessments in treatment pathways – either with behavioural modification strategies to target risk factors or with medications such as the incoming anti-amyloid therapies.

### Implications

Despite these limitations, our study demonstrates the potential of smartphone assessments as a tool for remotely tracking dementia risk and cognitive function in a large group of older adults. With the increasing number of dementia cases and the emergence of amyloid-targeting therapies, scalable dementia assessments are needed for risk identification, disease monitoring, treatment outcome tracking and recruitment for clinical trials. In addition, the high cost and risk associated with such therapies should focus efforts on secondary prevention of dementia. Multi-domain prevention programmes aimed at reducing dementia risk face logistical challenges in terms of scalability. Traditional in-person assessments are time-consuming, capacity-limited and resource-intensive. Accessible and reliable screening tools that track individuals over time, such as the smartphone app in this study, can overcome these limitations and offer the potential to alleviate the strain on healthcare systems, allocate resources and funding more efficiently, and identify individuals at the highest risk of dementia.

## Supporting information

Reid et al. supplementary material 1 Reid et al. supplementary material

Reid et al. supplementary material 2 Reid et al. supplementary material

Reid et al. supplementary material 3 Reid et al. supplementary material

Reid et al. supplementary material 4 Reid et al. supplementary material

Reid et al. supplementary material 5 Reid et al. supplementary material

Reid et al. supplementary material 6 Reid et al. supplementary material

Reid et al. supplementary material 7 Reid et al. supplementary material

Reid et al. supplementary material 8 Reid et al. supplementary material

Reid et al. supplementary material 9 Reid et al. supplementary material

Reid et al. supplementary material 10 Reid et al. supplementary material

Reid et al. supplementary material 11 Reid et al. supplementary material

## Data Availability

The analytic code associated with this study and the data that support the study findings are available from the corresponding author, G.R., on reasonable request.
