# Supplementary material for: The usability and reliability of a smartphone application for monitoring future dementia risk in ageing UK adults
Source: Br J Psychiatry. 2024 Jun;224(6):245–51. doi: 10.1192/bjp.2024.18 (PMC11443166; doi:10.1192/bjp.2024.18)
Supplement: Reid et al. supplementary material 1 — Reid et al. supplementary material [file S0007125024000187sup001.docx]

**Supplementary Material 1**

Full transcript of the Five Lives questionnaire assessment. The questionnaire is divided in three sections: “About you”, “Your health”, and “Your lifestyle”. Some questions also have an “info button” which the participant can tap to receive more information or clarification about the question.

| ****Module 1**** ****“About you”**** | | | |
| --- | --- | --- | --- |
|  | Question | Optional answers | info button |
| 1 | Please choose your date of birth. | Date picker |  |
| 2 | What is your sex? | Radio Buttons: **Male** \| **Female** \| **Other** |  |
| 3 | What is your highest level of education? | Radio Buttons: **No formal education** \| **Primary school** \| **Secondary school** \| **College (e.g. A levels)** \| **Bachelor’s degree or equivalent** \| **Postgraduate degree or equivalent** |  |
| 4 | What is your status in relation to work? | Radio Buttons: **I am still working** \| **I used to work and have now retired** \| **I am unemployed** |  |
| If for **4** the option selected = “**I am still working”** next question is: | | | |
| 4.1 | How many days per week do you work? | Picker: from **0** to **7** |  |
| If for **4.1** the option selected **>0** next question is: | | | |
| 4.1.1 | Do you do any shift work? | **Yes** \| **No** |  |
| Then regardless of the answer of **4.1** and **4.1.1** next question is: | | | |
| 4.1.2 | What is the highest level of occupation you have achieved so far? | Radio Buttons: **Junior** \| **Mid-level** \| **Senior** |  |
| If for **4** the option selected = “**I worked and now retired”** next question is: | | | |
| 4.2 | How many years did you work for? | Picker: from **0** to **100** |  |
| If for **4.2** the option selected **>0** next question is: | | | |
| 4.2.1 | How long ago was your last job? | Month and Year picker |  |
| Then regardless of the answer of **4.2** and **4.2.1** next question is: | | | |
| 4.2.2 | What is the highest level of occupation you achieved? | Radio Buttons: **Junior** \| **Mid-level** \| **Senior** |  |
| 5 | What is your height? | Slider:   - **cm**: from **55** to **270** - **ft**: **1’9** to **9’0** |  |
| 6 | What is your weight? | Slider:   - **kg**: from **25** to **200** - **st**: from **3st 13lb** to **31st 6lb** - **lb**: from **55** to **440** |  |
| 7 | What is your level of proficiency in English? | Radio Buttons: **Native** \| **Novice** \| **Intermediate** \| **Advanced** \| **Proficient** |  |
| 8 | How many languages do you speak fluently? | Radio Buttons: **1** \| **2** \| **3** \| **4** \| **5** \| **More than 5** |  |
| 9 | What best describes the area where you live: | Radio Buttons: **Urban** \| **Suburban** \| **Rural** |  |
| 10 | How do you think your memory is compared to others in your age group? | Radio Buttons: **Worse** \| **Similar** \| **Better** |  |
| 11 | How do you think your memory is compared to a few years ago? | Radio Buttons: **Worse** \| **Similar** \| **Better** |  |
| 12 | In the past four weeks, did you have any difficulties or require help with any of the following: | Multiple choice question  checkbox with a null option  (**We should not allow users to select 'None of the above' if any other option has been selected.**)  **Writing checks**  **Paying bills**  **Balancing a check book**  **Keeping track of events**  **Remembering appointments**  **Remembering family occasions**  **Remembering holidays**  **Remembering to take required medications**  **Remembering what happened in the past few days**  **Remembering names of familiar people**  **None of the above** | Tick all the relevant answers |
| 13 | Do you have a first degree relative that has been diagnosed with dementia or cognitive impairment? | Check box with null: **Yes, my father/Yes, my mother/Yes, my brother/Yes, my sister/Yes, a grandparent from my father’s side/Yes, a grandparent from my mother’s side/ No/ I don’t know** |  |
| If for 13 the option selected is different from **‘No’** or **'I don’t know'** next question is 13.1  else move to Module 2 | | | |
| 13.1 | Were they older or younger than 65 when diagnosed? | Radio Buttons: **Younger than 65** \| **Older than 65** |  |

| ****Module 2**** ****“Your health”**** | | | | |
| --- | --- | --- | --- | --- |
|  | Question | Optional answers | info button | |
| 1 | Do you know what your blood pressure is? | Radio Buttons and Slider tab:   - **Estimate** > Options: **Lower than normal** \| **Within normal range** \| **Higher than normal** \| **I don’t know** \| **Answer Later** - **Exact value** > Slider: from **50mm Hg** to **200 mm Hg** |  | |
| 2 | Do you know what your resting heart rate is? | Radio Buttons and Slider tab:   - **Estimate** > Options: **Lower than normal** \| **Within normal range** \| **Higher than normal** \| **I don’t know** \| **Answer Later** - **Exact value** > Slider: from **40 BPM** to **120 BPM** | BPM=beats per minute | |
| 3 | Have you ever experienced any heart-related issues? | **Yes** \| **No** |  | |
|  | If for **3** the option selected = **“Yes”** next question is **3.1**  else move to **4** | | | |
| 3.1 | Have you ever suffered from any of the following heart or blood-related issues? | Checkbox  [Multiple choice]  Heart attack  Cardiac arrest  Congestive heart failure  Hypertension  Atrial fibrillation  Peripheral vascular disease  Angina  Other | Tick all the relevant answers | |
| 3.2 | Have you ever gone through any of the following heart-related procedures? | checkbox with a null option  [Multiple choice]  **We should not allow users to select 'No' if any other option has been selected.**  Cardiac bypass procedure  Angioplasty  Endarterectomy  Stent  Other  No | Tick all the relevant answers | |
| 3.3 | Do you currently take any medications for your heart or blood condition/s? | **Yes** \| **No** |  | |
|  | If for **3.3** the option selected = **“Yes”** next question is **3.3.1**  else move to **4** | | | |
| 3.3.1 | Do you take anticoagulant or antiplatelet drugs? | **Yes** \| **No** | Anticoagulants and antiplatelet drugs eliminate or reduce the risk of blood clots. They're often called blood thinners, and they help prevent or break up dangerous blood clots that form in your blood vessels or heart. | |
| 4. | Do you know what your blood glucose level is? | Radio Buttons and Slider tab:   - Estimate Options: **Lower than normal** \| **Within normal range** \| **Higher than normal** \| **I don’t know** \| **Answer Later** - **Exact value** > Slider: from **2 mmol/L** to **10 mmol/L** |  | |
| 5 | Have you been diagnosed with diabetes? | **Yes** \| **No** |  | |
| If for **5** the option selected = **“Yes”** next question is **5.1**  else move to **6** | | | | |
| 5.1 | Do you currently take any medication for diabetes? | **Yes** \| **No** |  | |
| If for **5.1** the option selected = **“Yes”** next question is **5.1.1**  else move to **6** | | | | |
| 5.1.1 | Do you need to take tablets or insulin? | Radio Buttons: **Tablets** \| **Insulin** |  | |
| 6 | Do you need to take any medication for having high cholesterol? | **Yes \| Previously \| No** |  | |
| 7 | Do you know your total cholesterol level? | Radio Buttons and Text Fields: **- Estimate** > Options: **Lower than normal** \| **Within normal range** \| **Higher than normal** \| **I don’t know** \| **Answer Later** - **Exact value** > Text Fields (units mmol/L or mg/dL):  - Cholesterol (required) - HDL Cholesterol - LDL Cholesterol |  | |
| 8 | Have you ever suffered from a stroke? | **Yes** \| **No** |  | |
| 9 | Have you ever suffered from a mini-stroke (transient ischaemic attack)? | **Yes** \| **No** |  | |
| 10 | Have you ever been diagnosed with a vitamin B12 deficiency? | **Yes** \| **No** |  | |
| If for **10** the option selected = “**Yes”** next question is **10.1**  else move to **11** | | | | |
| 10.1 | Are you taking replacements for vitamin B12? | **Yes** \| **No** |  | |
| 11 | Have you ever been diagnosed with a thyroid disease? | **Yes** \| **No** |  | |
| If for **11** the option selected = “**Yes”** next question is **11.1**  else move to **12** | | | | |
| 11.1 | Are you taking thyroxine replacement medication? | **Yes** \| **No** |  | |
| 12 | Have you ever been diagnosed with a kidney problem? | **Yes** \| **No** |  | |
| If for **12** the option selected = “**Yes”** next question is **12.1**  else move to **13** | | | | |
| 12.1 | Are you on dialysis? | **Yes \| Previously \| No** |  | |
| 13 | Have you ever been diagnosed with an alcohol use disorder? | **Yes** \| **No** | Alcohol abuse — clinically significant impairment occurring over a 12-month period manifested in one of the following areas: work, driving, legal, or social | |
| 14 | Do you have any hearing problems? | **Yes** \| **No** |  | |
| If for **14** the option selected = **“Yes”** next question is **14.1**  else move to **15** | | | | |
| 14.1 | Is your hearing problem being treated? | **Yes** \| **No** | If you are using hearing aids that fix your hearing problems, you can answer “no” to this question. | |
| 15 | Do you have any problems with your sight? | **Yes** \| **No** | If you don’t have any visual problems, can see well using glasses or have undergone successful vision-correcting surgery, you can answer “no” to this question. | |
| If for **15** the option selected = **“Yes”** next question is **15.1**  else move to **16** | | | | |
| 15.1 | Is your sight problem being treated? | **Yes** \| **No** |  | |
| 16 | Are you colour blind? | **Yes** \| **No** |  | |
| 17 | Have you ever been diagnosed with a mood disorder? | **Yes** \| **No** |  | |
| If for **17** the option selected = **“Yes”** next question is **17.1** | | | | |
| 17.1 | Have you ever been clinically diagnosed with depression? | **Yes** \| **No** |  | |
| 18 | Have you been diagnosed with any psychiatric disorders? | **Yes** \| **No** |  | |
| If for 17 the option selected = **“Yes”** next question is **18.1** | | | | |
| 18.1 | Have you been diagnosed with any of the following? | **Checkbox with a null option**  [Multiple options]   - Schizophrenia - Psychosis - Bipolar disorder - Generalised anxiety disorder - Attention deficit hyperactivity disorder (ADHD) - Post-traumatic stress disorder (PTSD) - Obsessive compulsive disorder (OCD) - Personality disorder - Other | Tick all the relevant answers | |
| 19 | Have you been diagnosed with any of the following neurological diseases? | **Checkbox with a null option**  [Multiple options]   - Parkinson’s disease - Epilepsy - Multiple sclerosis - Migraine - Other neurological disease - Never diagnosed with any neurological disease | Tick all the relevant answers | |
| 20 | Have you ever had a traumatic brain injury (TBI)? [yes/no] | **Yes** \| **No** | TBI is a head injury that affects the brain. This can be from a hit or blow to the head (e.g. a concussion) or a penetrating injury, and can result in a period of unconsciousness, and/or feeling sick and dizzy. | |
| 21 | Have you ever had a head injury that caused a loss of consciousness for at least 30 minutes? | **Yes** \| **No** |  | |
| 22 | Have you been diagnosed with dyslexia? | **Yes** \| **No** |  | |
| 23 | Have you been diagnosed with inflammatory or irritable bowel disease? | **Yes** \| **No** |  | |
| 24 | Have you been diagnosed with any type of dementia? | **Yes** \| **No** |  | |
| 25 | Have you been diagnosed with mild cognitive impairment (MCI?) | **Yes** \| **No** |  | |
| 26 | Do you take any medication on a regular basis? | **Yes** \| **No** |  | |
|  | If for **26** the option selected = **“Yes”** next questions is **26.1 else go to 27** | | | |
| 26.1 | Please tick all of the medications you take on a regular basis: | Checkbox  [Multiple answer]  **Lipid lowering medications (usually used to reduce cholesterol levels)**  **Anti-inflammatory medications (usually used to relief pain)**  **Anti-hypertensives (usually used to lower blood pressure)**  **Antidepressants (usually used for a various psychiatric disorders)**  **Anxiolytic, sedative, or hypnotic medications (usually used to reduce emotional tension or anxiety)**  **Antiparkinsonian medications (used for Parkinson’s disease or parkinsonism)**  **Blood glucose medications**  **Other** | Tick all the relevant answers | |
|  | For question **27**, time taken by the user to answer the question should be recorded | | | |
| 27 | Without checking, do you know what today's date is? If you don’t know, make your best guess. | Day, Month and Year picker |  | |
| 28 | Do you have any medical problems that may affect how well you type? | Yes \| No |  | |
| If for **28** the option selected = **“Yes”** next questions is **28.1** else **complete this session** | | | | |
| 28.1 | Which of the conditions below are the cause of the typing issue? | | Checkboxes  [Multiple answer]   - Arthritis Injury - Tremor - Eyesight problems - Dexterity - Neurological disorders - Other |  |

| **Module 3** **“Your lifestyle”** | | | |
| --- | --- | --- | --- |
|  | Question | Answers | Info button |
| 1 | On average, how much time do you spend exercising per week?  Moderate  e.g. walking, hiking, swimming, etc.  Intense  Exercise that makes it difficult for you to speak, e.g. running, boxing, etc.  Mixed  Mixed = a mix between moderate and intense training | Radio Buttons: **I don’t exercise** \| **Less than one hour** \| **1-2 hours** \| **2-3 hours** \| **More than 3 hours** |  |
|  | If for **1** the option selected =! (different from) **“I do not exercise”** next question is **2** else move to **1.1** | |  |
| 1.1 | How would you define the intensity of your training? | Radio Buttons:   - **Moderate** (e.g. walking, hiking, swimming, etc.) - **Intense** Exercise that makes it difficult for you to speak, e.g. running, boxing, etc. - **Mixed** = a mix between moderate and intense training |  |
| 2 | Do you smoke? | Radio Buttons:  **I am a current smoker/ I used to smoke but I quit/ Never smoked** | Being a smoker means you smoke any tobacco product, either daily or occasionally |
| If for **2** the option selected =! (different from) **“Never smoked”** next question is **2.1** else move to **3** | | | |
| 2.1 | On average, how many cigarettes do/did you smoke a day? | Radio buttons:  **Less than 5 \| 5-9 \|10-14 \|15-24 \|25 or more** |  |
| 3 | Please estimate how much time you meet friends and family for social interactions on a typical week. | Radio buttons:  **None \| Up to 1 hour \| 1-3 hours \| 4-6 hours \| 7-10 hours \| More than 10 hours** |  |
| 4 | Please rate how lonely you feel | Slider scale: from 01 to 10   - 01 \| Not Lonely - 05 \|   Quite Lonely   - 10 \| Very Lonely |  |
| 5 | Over the past week, how many hours of sleep did you have each night on average? | Picker: from **1 hour** to **12 or more** |  |
| 6 | Over the past week, how sleepy have you felt during the day? | Slider scale: from 01 to 10  [Slider Scale / Range: 1-10 / Colour: Cosy Blue]   - 01 \| Not Sleepy - 05 \| Quite Sleepy - 10 \| Really Sleepy |  |
| 7 | Do you have any regular problems with your sleep? | **Yes** \| **No** |  |
| If for **7** the option selected = **“Yes”** next question is **7.1**  else move to **8** | | |  |
| 7.1 | Please select the issue/s you are experiencing: | Multiple choice question  **Difficulty falling asleep** **\| Difficulty staying asleep** \| **Waking up too early** \| **Violent movements during sleep \| Insomnia \| Sleep apnea \| Other** |  |
| 8 | How many portions of fruit and vegetables do you eat on a typical day? | Radio Buttons: **0 \|** **1** \| **2** \| **3** \| **4** \| **5** \| **More than 5** | A portion of fruit is approximately 80g. This counts as approximately 2 small fruit (e.g. 2 plums or satsumas), 1 medium fruit (e.g. 1 apple) or half of a large fruit (e.g. half of a grapefruit). |
| 9 | Do you typically eat at least two portions of fish a week? | **Yes** \| **No** | A portion of fish is approximately 140g (4.9oz). |
| 10 | Do you usually eat a handful of nuts on a typical day? | **Yes** \| **No** |  |
| 11 | How many units of alcohol do you drink on a typical week? | **Cards** with **number selector**: Small glass of red / white / rosé wine \| 125ml, 12%   - Standard glass of red / white / rosé wine \| 175ml, 12% - Large glass of red / white / rosé wine \| 250ml, 12% - Bottle of lager / beer / cider \| 330ml, 5% - Can of lager / beer / cider \| 440ml, 5.5% - Pint of lower-strength lager / beer / cider \| 3.6% - Pint of higher-strength lager / beer / cider \| 5.2% - Single small shot of spirits \| 25ml, 40% - Large single measure of spirits \| 35 ml 40% - Alcopop \| 275ml, 5.5% |  |
| 12 | How many glasses of water do you drink on a typical day? | Radio Buttons: **3 or less** \| **4-6** \| **7-9** \| **10-12** \| **13-15** \| **16 or more** |  |
| 13 | Have you ever carried out mixing, applying or loading of any herbicides, pesticides, fumigants, weed killers or fungicides? | Radio Buttons: **Yes \| No \| I don’t know** |  |
| 14 | How much time do you spend reading for leisure during a typical week (e.g. books, newspapers)? | Radio Buttons: **No time at all** \| **Less than one hour per week** \| **Between 1-3 hours per week** \| **More than 3 hours per week** | This can include either online (soft copy) or offline (hard copy) reading. |
| 15 | How much time do you spend completing mentally stimulating activities (e.g. puzzles, crosswords, logic) during a typical week? | Radio Buttons: **No time at all** \| **Less than one hour per week** \| **Between 1-3 hours per week** \| **More than 3 hours per week** | This can include games played both online and offline. |
| 16 | How much time do you spend playing a musical instrument during a typical week? | Radio Buttons: **No time at all** \| **Less than one hour per week** \| **Between 1-3 hours per week** \| **More than 3 hours per week** |  |
| 17 | How much time do you spend learning/practising a new skill during a typical week? For example, learning a new language. | Radio Buttons: **No time at all** \| **Less than one hour per week** \| **Between 1-3 hours per week** \| **More than 3 hours per week** |  |
| 18 | On a typical week, please rate how easy it is for you to feel relaxed. | Slider scale:  [Slider Scale / Range: 1-10 / Colour: Harmonious Yellow]   - 01 \| Very hard - 10 \| Very easy |  |
| 19 | Please rate your mood on a typical week. | Slider scale: from 01 to 10  [Slider Scale / Range: 1-10 / Colour: Harmonious Yellow]   - 01 \| Very upset - 10 \| Very happy |  |
| 20 | On a typical week, please rate how much you enjoy your day-to-day activities. | Slider scale: from 01 to 10  [Slider Scale / Range: 1-10 / Colour: Harmonious Yellow]   - 01 \| I rarely enjoy them - 10 \| I mostly enjoy them |  |
| 21 | How likely are you to work on improving your lifestyle (physical activity, sleep, diet, mental stimulation, stress & mood) in the short term? | Slider scale: from 01 to 10  [Slider Scale / Range: 1-10 / Colour: Calming Dark Blue]   1. 01 \| Very unlikely 2. 10 \| Very likely |  |
| 22 | How likely are you to discuss your brain health with your doctor? | Slider scale: from 01 to 10  [Slider Scale / Range: 1-10 / Colour: Calming Dark Blue]   1. 01 \| Very unlikely 2. 10 \| Very likely |  |
