# Supplementary figures and images for: The usability and reliability of a smartphone application for monitoring future dementia risk in ageing UK adults
Source: Br J Psychiatry. 2024 Jun;224(6):245–51. doi: 10.1192/bjp.2024.18 (PMC11443166; doi:10.1192/bjp.2024.18)

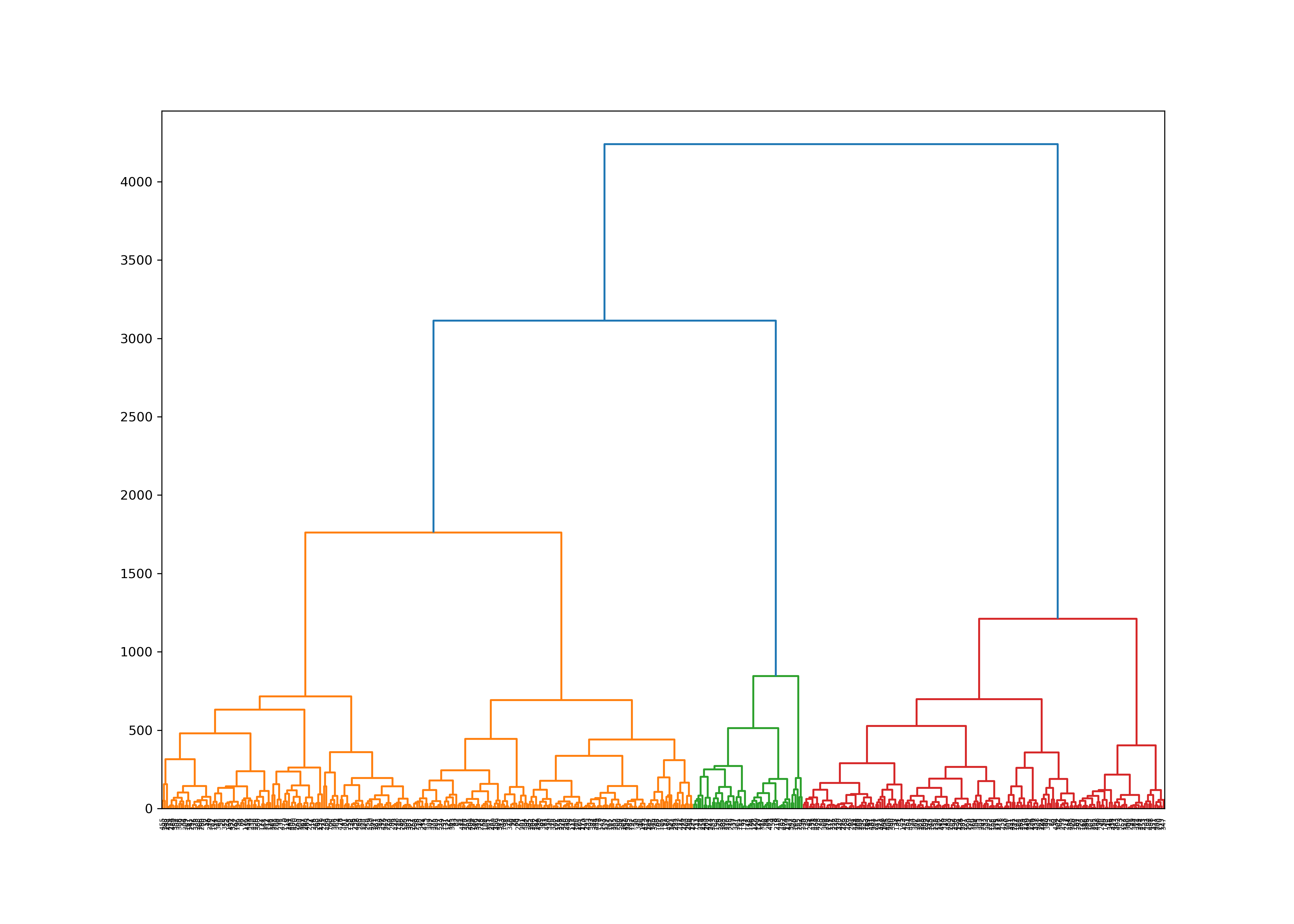

Supplement: Reid et al. supplementary material 2 — Reid et al. supplementary material [file S0007125024000187sup002.zip › supplementary_material_10.png]

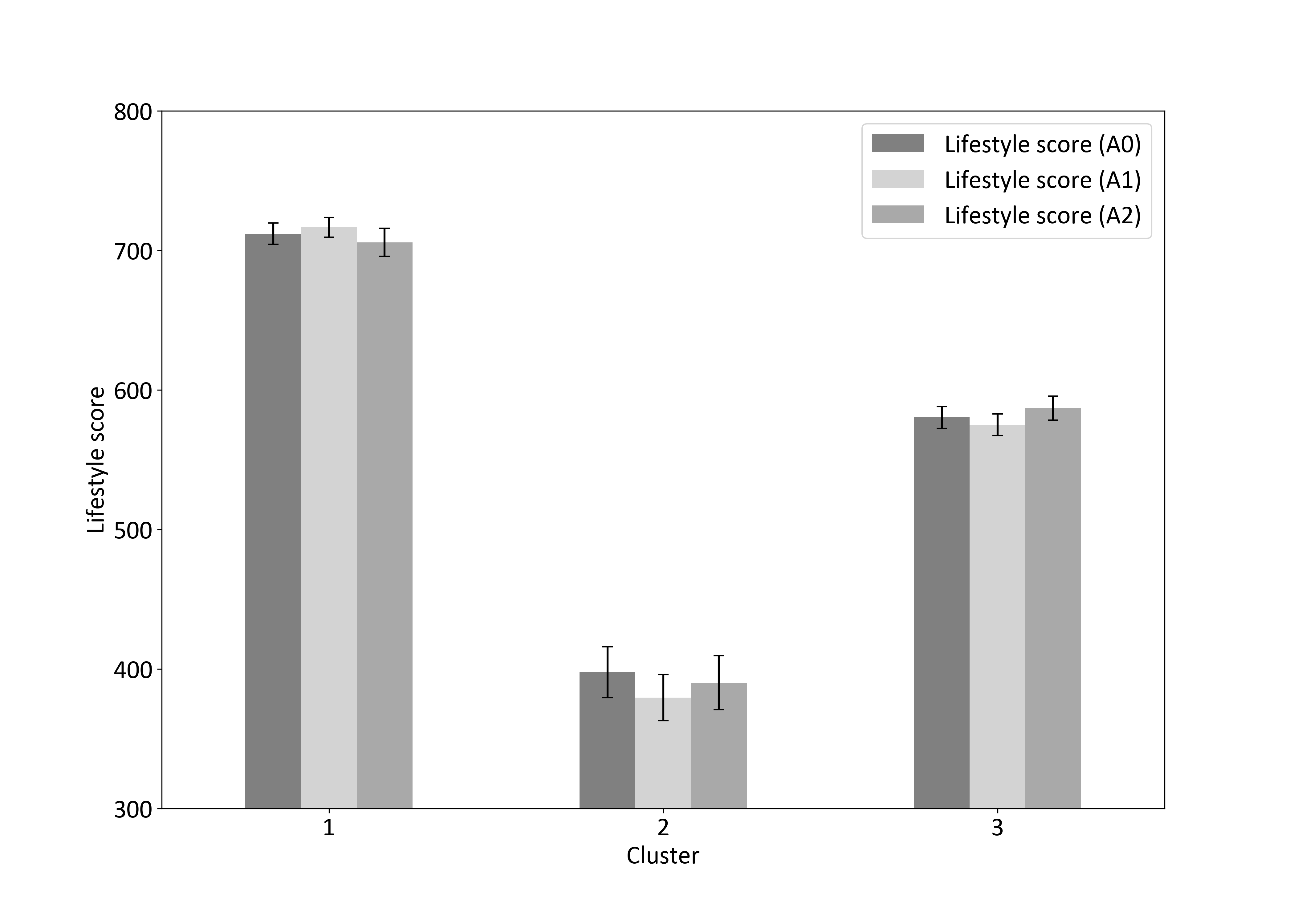

Supplement: Reid et al. supplementary material 3 — Reid et al. supplementary material [file S0007125024000187sup003.zip › supplementary_material_11.png]

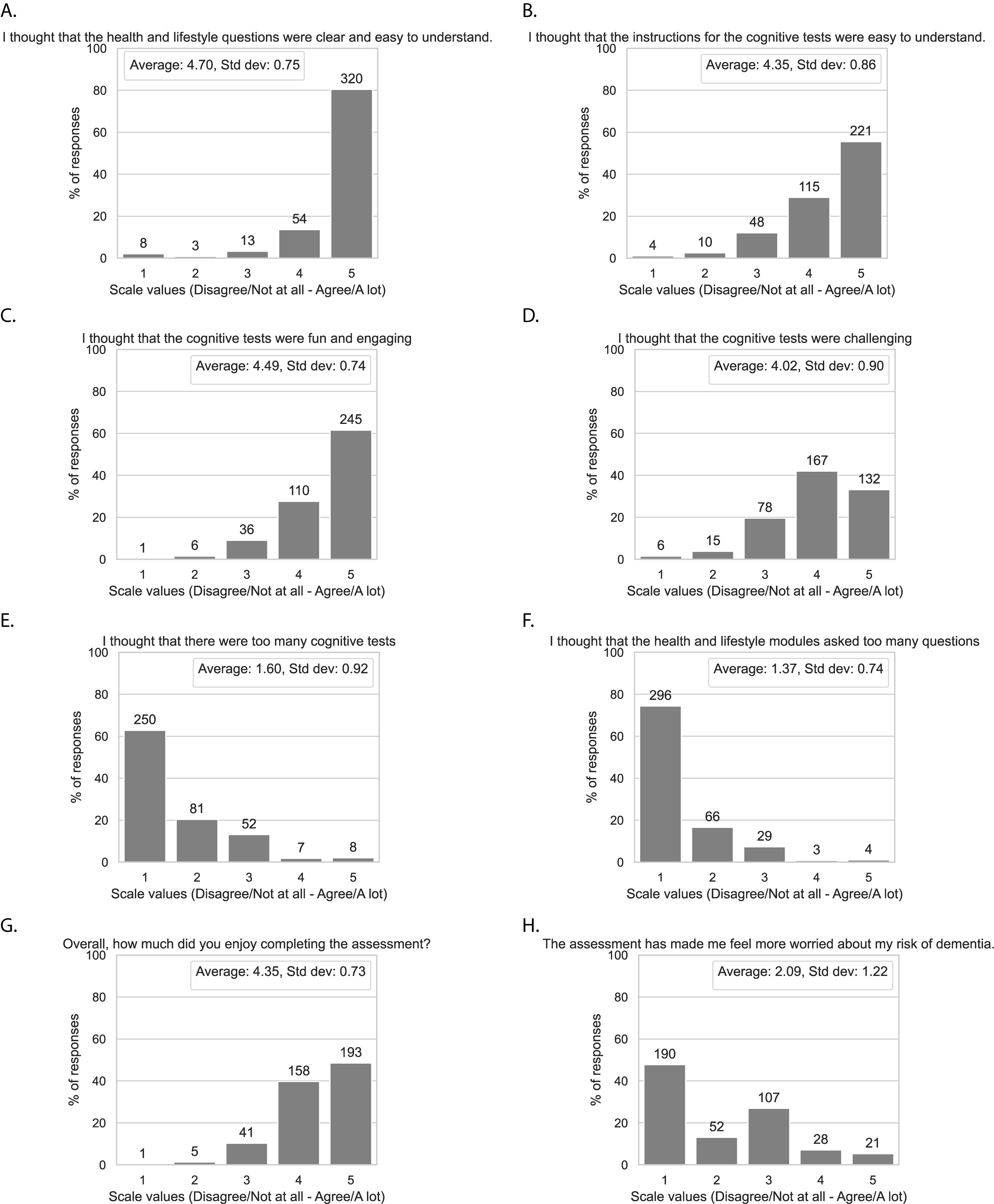

Supplement: Reid et al. supplementary material 8 — Reid et al. supplementary material [file S0007125024000187sup008.zip › supplementary_material_6.tiff]

**Supplementary Material 9**

*A Scatter Plot of the Correlation between CANTAB-PAL and Cast Scores*


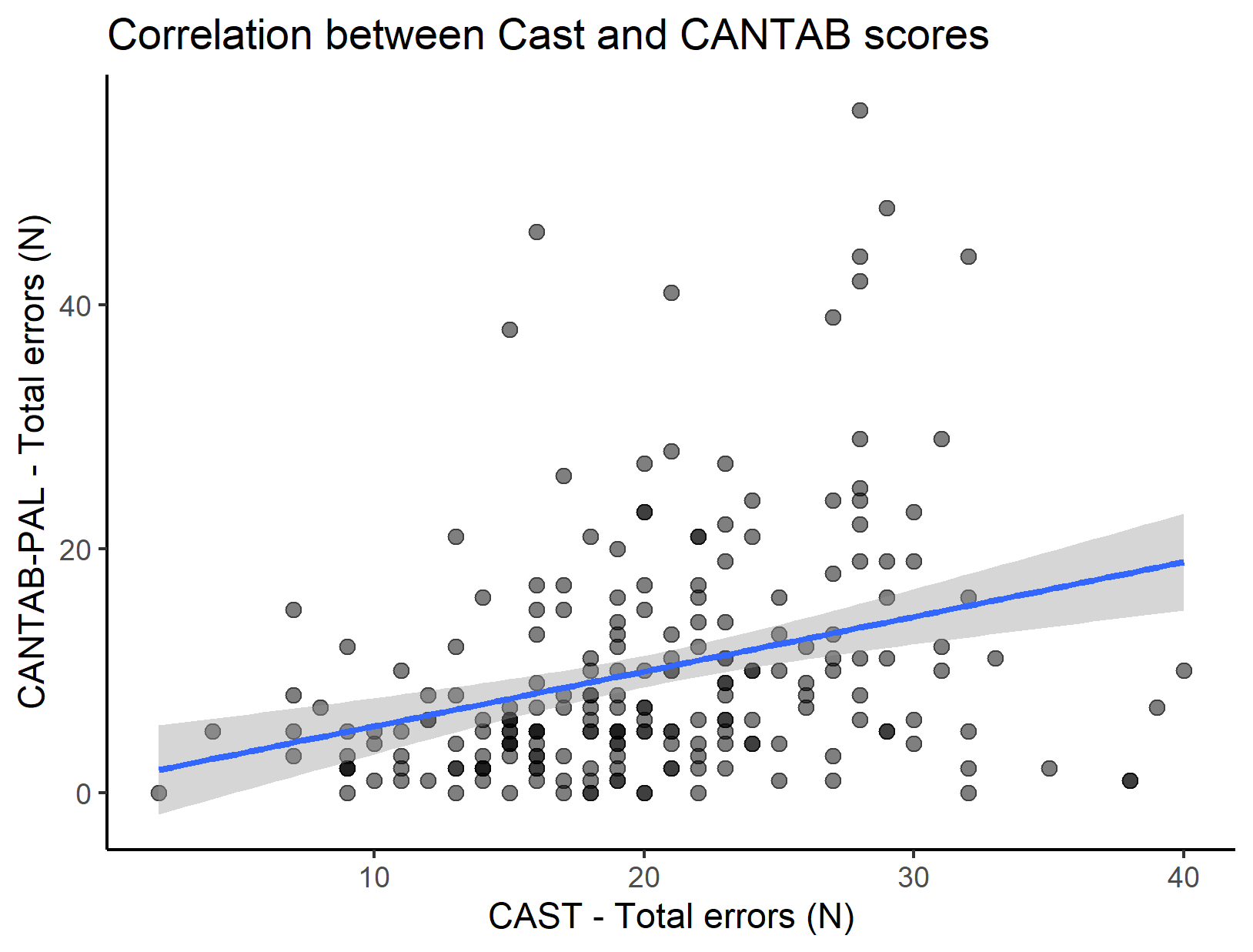

Supplement: Reid et al. supplementary material 11 — Reid et al. supplementary material [file S0007125024000187sup011.docx]
