# Supplementary material for: The usability and reliability of a smartphone application for monitoring future dementia risk in ageing UK adults
Source: Br J Psychiatry. 2024 Jun;224(6):245–51. doi: 10.1192/bjp.2024.18 (PMC11443166; doi:10.1192/bjp.2024.18)
Supplement: Reid et al. supplementary material 4 — Reid et al. supplementary material [file S0007125024000187sup004.docx]

# **Supplementary Material 2**

# This document contains the representative screen capture images showing the design and user interface for each of the Five Lives’ cognitive tests. It also contains more detailed descriptions of the tests and the theory and studies upon which each is based

# **Snap:** Verbal fluency

##
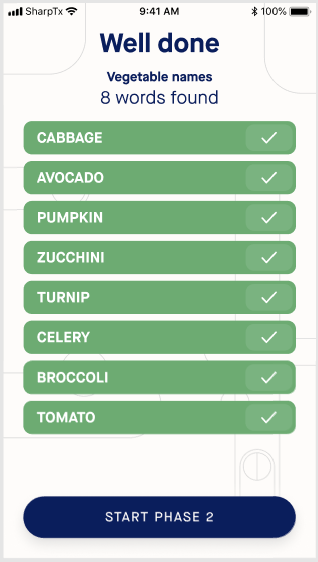

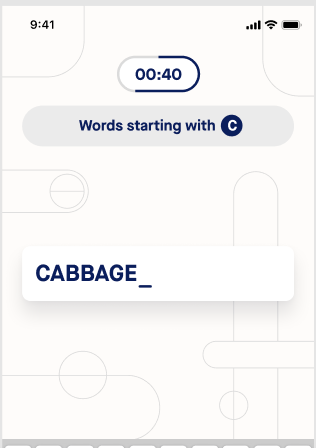
 The images show an example of a Snap round where participants have to enter words starting with the letter “C”.

# **Breeze:** Verbal memory


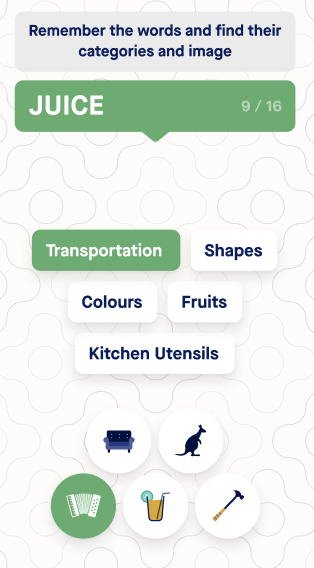

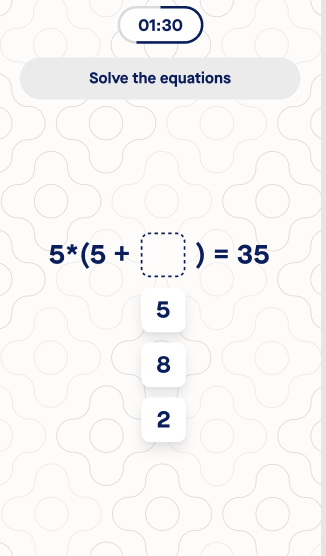

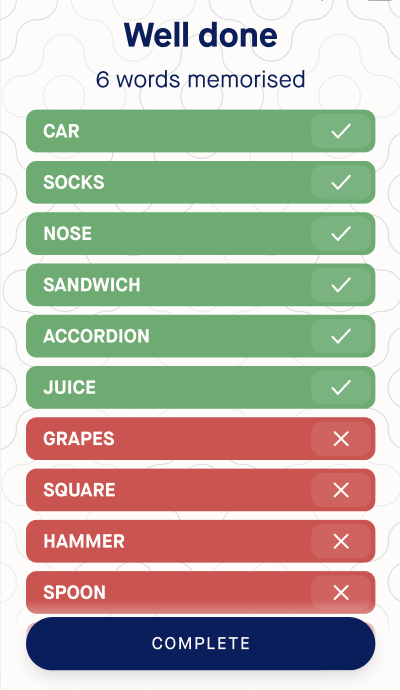


The three images represent the three stages of the Breeze game from left to right: learning phase, math task and recall phase.

# **Cast**: Spatial and numerical memory


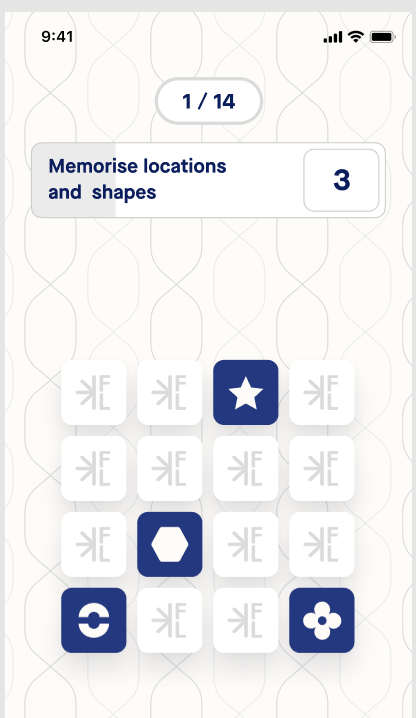


This image shows an example of a 4x4 grid with 4 shapes to be memorised by the participant.

#

# **Swift**: Executive Function


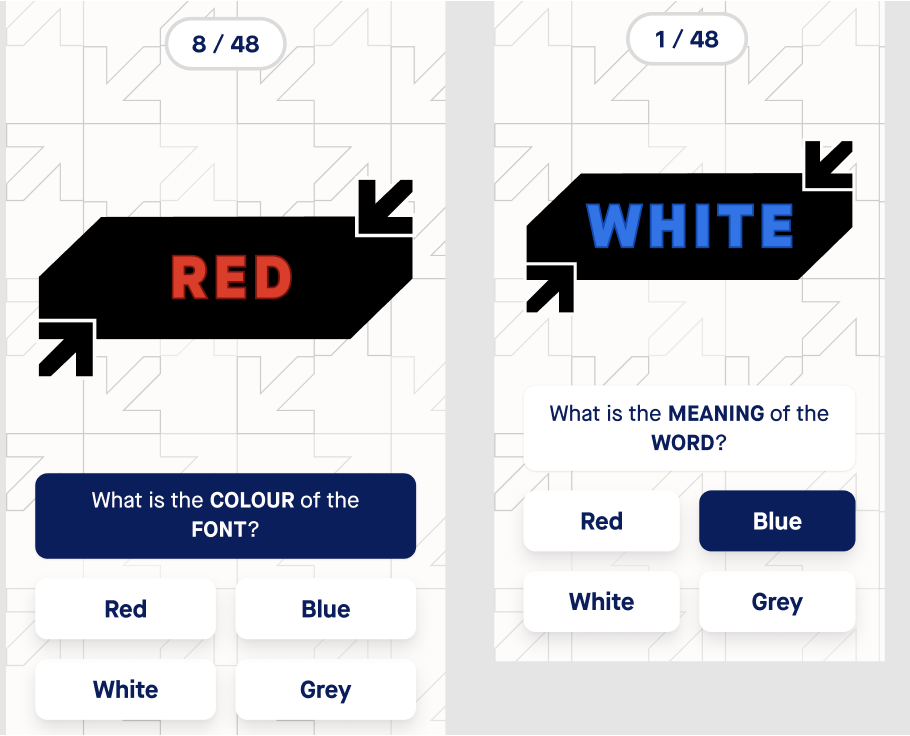


# The images show a congruent (left) and an incongruent (right) stimulus in the Swift cognitive test.

# **Twist:** Speed of processing

#
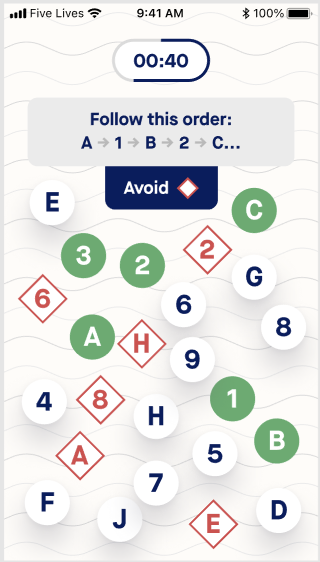

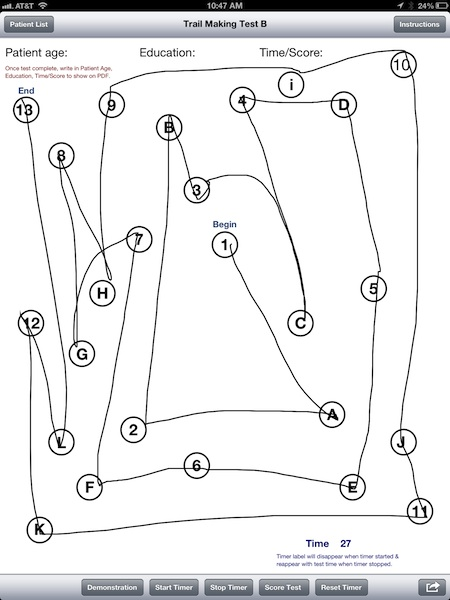
The images show a possible solution of a number and letter trail in Twist (left) and an actual screen capture of a Twist round with distractor shapes in red (right).

**Snap - verbal fluency.** Snap measured two types of verbal fluency: category and letter fluency. Participants were asked to type as many words from a specific category (semantic fluency) or starting with a specific letter (phonemic fluency) as possible. The categories used for prompting were “animals”, “vegetables' ', "fruits ","words starting with F”, and “words starting with C”. Performance in Snap was measured as the number of words produced in response to a prompt.

**Breeze - verbal memory and numerical skills.** Breeze is based on the Free and Cued Recall Test [(Buschke, 1984; Grober et al., 1992; Lemos et al., 2014)](https://paperpile.com/c/gvfcB3/AiFPg+6x7lg+Z1Q7Y). The test has three phases: a learning phase, maths tasks, and a recall phase. In the learning phase, participants were asked to memorise 16 different words (presented one after the other) picked from the following categories, for later recall: animals, body parts, clothing, colours, drinks, emotions/feelings, food, fruits, furniture, kitchen utensils, musical instruments, shapes, sports, tools, transportation, vegetables, places. The participants were also asked to allocate the category and to match an image to the word being memorised. Following the learning phase, participants were given a time-based distraction task involving mathematical operations to prevent rehearsal of words before recall. This distraction phase lasted for 60 seconds and involved a series of two-digit addition and subtraction operations. Participants were presented with equations such as "34+29" or "52-17" and were required to select the correct answer from four possible options. In the recall phase, participants were given 90 seconds to type as many words as possible from memory (free recall). For words not recalled, participants were given category cues (cued recall). Performance on Breeze was measured as the number of correctly recalled words in the free and cued recall phases.

**Cast - spatial and visual memory**. The Five Lives cognitive test Cast was designed as an analog of The Paired Associates Learning (PAL) task [(Barnett et al., 2016)](https://paperpile.com/c/gvfcB3/JLqI). It measured spatial and visual memory by requiring participants to recall the positions of different visual shapes on a grid of white squares. First, participants were shown the positions of several shapes on the grid for 5 seconds. Then, the shapes were hidden and participants were instructed to recall the location of each “target” shape presented at the top of the screen, following a specific order. The difficulty of the test was progressively increased by increasing the size of the grid (from 4 x 4 to 5 x 5), the number of shapes to remember (between 2 and 6), and the number of rounds with each combination of grid size and number of shapes. No feedback was provided between rounds.

This cognitive test was redeveloped during the course of the study to include more difficult levels after preliminary analyses discovered most participants were able to successfully complete the cognitive test because it was too easy. This resulted in a lower sample size for this cognitive test after the data from the easier version were excluded. Performance on this cognitive test was measured as the number of correct shape locations identified on the first attempt. The maximum number of correct shape locations to be remembered across all trials was 46.

**Swift - executive functions.** The Swift cognitive test is analogous to the extensively validated Stroop task [(MacLeod, 2005)](https://paperpile.com/c/gvfcB3/nwVN). In this test participants were shown colour names written in either matching colours (e.g. RED written in red) or mismatching colours (e.g. RED written in blue): referred to as “congruent” and “incongruent” stimuli respectively. Participants were then asked to choose the colour of the word or its meaning by tapping on one of four possible answers. Task performance was scored as median reaction time for correct answers on (i) congruent stimuli and (ii) incongruent stimuli.

**Twist - speed of processing and cognitive control.** The cognitive test Twist was based on the Trail Making Test [(Bowie & Harvey, 2006)](https://paperpile.com/c/gvfcB3/Cfx7q). In this test participants were asked to draw a line connecting elements (numbers and/or letters) in consecutive order according to specified rules. There were three levels of trail difficulty: level 1 (sequence: A-B-C-D-E-F-G…), level 2 (sequence: A-1-B-2-C-3-D-4-E-5…) and level 3 (sequence: A-1-B-2-C-3-D-4-E-5… + distracting red rhomboids containing letters and numbers). Performance was measured as the time taken to complete the task. Errors were incorporated into response time as the participant was not allowed to progress until they connected to the correct element.
