# Supplementary material for: The usability and reliability of a smartphone application for monitoring future dementia risk in ageing UK adults
Source: Br J Psychiatry. 2024 Jun;224(6):245–51. doi: 10.1192/bjp.2024.18 (PMC11443166; doi:10.1192/bjp.2024.18)
Supplement: Reid et al. supplementary material 5 — Reid et al. supplementary material [file S0007125024000187sup005.docx]

**Supplementary Material 3**

The sleep pillar score was summed from hours of sleep, tiredness level and sleep problems, where:

- **Hours of sleep:** For ≤ 7 hours of sleep, the score was obtained by multiplying sleep hours by 47.61. For 8 hours of sleep, the score was set at 285.7. For > 8 hours of sleep, the score was calculated as (12 - sleep hours) multiplied by 47.61.
- **Tiredness level:** The score was computed as (10 - tiredness_level) multiplied by 37.01, where *tiredness level* was measured on a 10-point Likert scale.
- **Sleep problems:** If any sleep problems were reported, the score was set to 0; otherwise, the score was 333.33.

The other pillar scores were calculated as follows:

- **Physical activity score** = (exercise intensity + duration * 3) * 83.33
- **Stress and mood score** = relaxation rating * 27.78 + mood rating * 27.78 + enjoyment of activities multiplied by 27.78 + (10 - loneliness score) * 27.78
- **Mental stimulation score** = time reading * 66.6 + time mental stimulation * 66.6 + time instrument * 66.6 + time skill * 66.6 + sociaI interactions * 5
- **Diet score =** fruit and vegetable portions * 40 + fish portions * 200 + nut portions * 200 + (200 - (alcohol units * 14.28; with alcohol units > 14 as 0)
