# Supplementary material for: The usability and reliability of a smartphone application for monitoring future dementia risk in ageing UK adults
Source: Br J Psychiatry. 2024 Jun;224(6):245–51. doi: 10.1192/bjp.2024.18 (PMC11443166; doi:10.1192/bjp.2024.18)
Supplement: Reid et al. supplementary material 6 — Reid et al. supplementary material [file S0007125024000187sup006.docx]

**Supplementary Material 4**

|  | | Category | | | Count | | | Percentage | |
| --- | --- | --- | --- | --- | --- | --- | --- | --- | --- |
| Participant sex | | Female | | | 510 | | | 67.46 | |
|  |  | Male | | | 246 | | | 32.54 | |
| Education level | | Postgraduate | | | 219 | | | 28.97 | |
|  |  | Undergraduate | | | 276 | | | 36.51 | |
|  |  | College | | | 143 | | | 18.92 | |
|  |  | Secondary | | | 116 | | | 15.34 | |
|  |  | No formal education | | | 2 | | | 0.26 | |
| Work status | | Retired | | | 504 | | | 66.67 | |
|  |  | Working | | | 239 | | | 31.61 | |
|  |  | Unemployed | | | 13 | | | 1.72 | |
| Area of residence | | Rural | | | 336 | | | 44.44 | |
|  |  | Suburban | | | 308 | | | 40.74 | |
|  |  | Urban | | | 112 | | | 14.81 | |
| Memory compared to others  (self-perceived) | | Similar | | | 625 | | | 82.67 | |
|  |  | Worse | | | 67 | | | 8.86 | |
|  |  | Better | | | 64 | | | 8.47 | |
| Memory compared to a few years ago  (self-perceived) | | Worse | | | 391 | | | 51.72 | |
|  |  | Similar | | | 358 | | | 47.35 | |
|  |  | Better | | | 7 | | | 0.93 | |
| Smoking status | | Non-smoker | | | 477 | | | 63.10 | |
|  |  | Smoker | | | 261 | | | 34.52 | |
|  |  | Ex-smoker | | | 18 | | | 2.38 | |
| Alcohol use disorder diagnosis | | Absent | | | 747 | | | 98.81 | |
|  |  | Present | | | 9 | | | 1.19 | |
|  | **Count** | | **Mean** | **Standard Deviation** | | **Median** | **25th Percentile** | | **75th Percentile** |
| Age | 756 | | 65.21 | 7.86 | | 66.00 | 60.00 | | 70.25 |
| Weight (kg) | 756 | | 75.51 | 16.27 | | 73.00 | 64.00 | | 85.00 |
| Height (cm) | 756 | | 167.78 | 9.11 | | 166.00 | 162.00 | | 174.25 |
