# Supplementary material for: The usability and reliability of a smartphone application for monitoring future dementia risk in ageing UK adults
Source: Br J Psychiatry. 2024 Jun;224(6):245–51. doi: 10.1192/bjp.2024.18 (PMC11443166; doi:10.1192/bjp.2024.18)
Supplement: Reid et al. supplementary material 7 — Reid et al. supplementary material [file S0007125024000187sup007.docx]

**Supplementary Material 5**

|  | Category | Count | Percentage |
| --- | --- | --- | --- |
| Has a relative with dementia | No | 318 | 65.57 |
|  | Yes | 167 | 34.43 |
| Relative older than 65 when diagnosed? | Yes | 150 | 89.82 |
|  | No | 17 | 10.18 |
| Blood pressure (estimate) | Average | 286 | 67.77 |
|  | High | 66 | 15.64 |
|  | Unknown | 40 | 9.48 |
|  | Low | 30 | 7.11 |
| Resting heart rate (estimate) | Average | 251 | 67.65 |
|  | Unknown | 71 | 19.14 |
|  | Low | 40 | 10.78 |
|  | High | 9 | 2.43 |
| Cholesterol (estimate) | Unknown | 210 | 45.16 |
|  | Average | 178 | 38.28 |
|  | High | 69 | 14.84 |
|  | Low | 8 | 1.72 |
| Diabetes diagnosis | Absent | 458 | 94.43 |
|  | Present | 27 | 5.57 |
| Had stroke | No | 478 | 98.56 |
|  | Yes | 7 | 1.44 |
| Had mini stroke | No | 469 | 96.70 |
|  | Yes | 16 | 3.30 |
| Vitamin B12 deficiency | No | 460 | 94.85 |
|  | Yes | 25 | 5.15 |
| Thyroid disease diagnosis | Absent | 434 | 89.48 |
|  | Present | 51 | 10.52 |
| Renal problem | Absent | 453 | 93.40 |
|  | Present | 32 | 6.60 |
| Hearing problems | Absent | 346 | 71.34 |
|  | Present | 139 | 28.66 |
| Visual problems | Absent | 272 | 56.08 |
|  | Present | 213 | 43.92 |
| Colour blindness | Absent | 478 | 98.56 |
|  | Present | 7 | 1.44 |
| Personality disorder | Absent | 16 | 88.89 |
|  | Present | 2 | 11.11 |
| Other psychiatric diagnosis | Absent | 15 | 83.33 |
|  | Present | 3 | 16.67 |
| Parkinson's disease | No | 483 | 99.59 |
|  | Yes | 2 | 0.41 |
| Epilepsy | No | 479 | 98.76 |
|  | Yes | 6 | 1.24 |
| Multiple sclerosis | No | 483 | 99.59 |
|  | Yes | 2 | 0.41 |
| Migraines | No | 408 | 84.12 |
|  | Yes | 77 | 15.88 |
| Other neurological diseases | No | 473 | 97.53 |
|  | Yes | 12 | 2.47 |
| Head injury | No | 468 | 96.49 |
|  | Yes | 17 | 3.51 |
| Loss of consciousness | No | 472 | 97.32 |
|  | Yes | 13 | 2.68 |
| Dyslexia | No | 445 | 99.11 |
|  | Yes | 4 | 0.89 |
| Bowel disease/IBS | No | 422 | 87.01 |
|  | Yes | 63 | 12.99 |
